# The aftermath of multiple trauma on a nation: unraveling Lebanon’s unique mental health struggle

**DOI:** 10.3389/fpsyt.2024.1444245

**Published:** 2025-01-14

**Authors:** Elie G. Karam, Mariam El-Jamal, Rayane Osman, Sana Toukan, Ghiwa Ishac Mouawad, Josleen Al Barathie

**Affiliations:** ^1^ Institute for Development, Research, Advocacy, and Applied Care (IDRAAC), Beirut, Lebanon; ^2^ Department of Psychiatry and Clinical Psychology, Saint George University of Beirut, Beirut, Lebanon; ^3^ Department of Psychiatry and Clinical Psychology, St George Hospital University Medical Center, Beirut, Lebanon; ^4^ Research Department, Ipsos SAL, Beirut, Lebanon

**Keywords:** prevalence, PTSD, depression, anxiety, national study

## Abstract

**Objective:**

This study examines the national prevalence of mental health disorders and their associated factors in Lebanon, specifically in the aftermath of the 2020 events, including the catastrophic events of Beirut blast and the concurrent financial meltdown amid the global pandemic.

**Methods:**

Conducted between July and September 2022, the study interviewed a nationally representative sample of 1,000 Lebanese via telephone, using the Computer Assisted Telephone Interview (CATI) system. Gender-specific bivariate and multivariate models were generated for probable posttraumatic stress disorder (PTSD), depression, and anxiety.

**Results:**

High rates of mental health disorders emerged — 47.8% screened positive for probable depression, 45.3% for probable anxiety, and 43.5% met the probable diagnosis for PTSD. Multivariate gender-specific analyses revealed no significant associations with governorate, employment status, or marital status, while the financial composite score consistently influenced all disorders.

**Conclusion:**

Lebanon faces a severe mental health crisis, evidenced by elevated rates of probable depression, anxiety, and PTSD. The universal impact of multiple traumas transcends typical determinants, emphasizing the need for nuanced interventions and targeted policy considerations.

## 1 Introduction

In recent years, Lebanon, now a lower-income Middle Eastern country, has experienced its worst combination of crises in history. In 2020, the Lebanese were concomitantly hit by a dual internal trauma: the financial meltdown and the Beirut Port Blast. The Beirut Port Blast was the largest non-nuclear blast recorded in modern history and resulted in many fatalities and thousands of destroyed dwellings and displaced families (1). Moreover, in 2020 the value of the Lebanese currency started deteriorating and by 2022 it lost more than 98 percent of its pre-crisis value with an average inflation of 171.2%—one of the highest rates globally (2). Both these traumas happened concurrently during the COVID-19 pandemic.

These multifaceted challenges have not only inflicted immense losses on the material front, but they have also given rise to a pressing concern—mental health disorders. When countries experience major adversities, including economic crises and disasters, mental health disorders become more pronounced and impede any recovery on the individual and societal levels (3–5). In the aftermath of disasters, whether natural or man-made, the rates of mental health disorders increase both within and outside the immediate vicinity of the trauma (5). In 2014, China experienced a series of Ammonium Nitrate explosions, which led to injuries and significant loss of life (6). Among the 2,395 participants directly exposed to the explosion, nearly 17% displayed symptoms of moderate to severe PTSD (7). In the United States, after the September 11 terrorist attack, a nationally representative cross-sectional study surveyed 2,273 adults found that PTSD affected individuals both directly impacted by the attack and those residing outside of New York. The prevalence of PTSD was 11.2% in New York, 2.7% in Washington, 3.6% in major metropolitan areas, and 4.0% in the rest of the country—one to two months after the attacks (8). The far-reaching effect of trauma was further underscored in a longitudinal study that revealed an incidence of 17% of probable PTSD among individuals residing outside New York two months after the attacks (9).

In parallel to the Beirut Port Blast, Lebanon was witnessing a financial collapse. The literature is rich with evidence of how such events can exacerbate mental health disorders. For example, following the recession of 2008 in Hong Kong, there was a rise in major depressive episodes (MDEs) that reached 20.1% among individuals with significant investment losses (10). Similarly in 2009, the economic recession in Greece resulted in a twofold increase in MDEs of the nationally studied population (11). Meanwhile, in Venezuela, a country grappling with a severe economic crisis, individuals with poor and extremely poor socioeconomic status were respectively 2.0 and 2.8 times more likely to exhibit anxiety symptoms, and 2.5 and 4.4 times more likely to display depressive symptoms compared to those with higher socioeconomic status (12). These findings offer significant insights into the mental health situation in Lebanon, shedding light on the potential challenges the country has faced, particularly in the aftermath of the banking crisis, which experts have likened to a Ponzi scheme (13, 14).

Concurrent with these events, as of August 21, 2022, COVID-19 has had a profound global impact, registering 593 million confirmed cases and 6.4 million reported deaths worldwide (15). Beyond the physical health toll, the literature consistently underscores the pandemic’s profound impact on global mental health, manifesting in heightened rates of anxiety and depression. For instance, PHQ-9 depression rates surged from 8.5% pre-pandemic to 27.8% post-pandemic in the United States (16). This trend was evident also in many other areas such as Peru, where depression scores quintupled from 6.4% in 2018 to 35% in 2020 (17). Other national studies echo these findings, with anxiety rates reaching up to 27.8% in Mexico by 2020 (18).

Given the unique situation in Lebanon, many have suspected that the mental health of the Lebanese to be severely affected (19). Unfortunately, to date, we do not have nationally representative studies that have explored this complex landscape. The only studies at our disposal are either collected their data through convenient samples from social media (20, 21), or investigated mental health outcomes within small, restricted subpopulation groups such as university students (22) or hemodialysis patients (23). None of the studies we are aware of evaluated the mental health of the Lebanese population on a national level. The latest nationwide study of this kind was conducted in 2006 in the context of the World Mental Health survey project (24) well before the calamities converged in the country. The 2006 surveys were face-to-face interviews utilizing the Composite International Diagnostic Interviews (CIDI), a well-known established fully structured diagnostic interview to assess disorders and treatment. They have contributed in shaping the mental health strategy of the country and served as benchmarks for many other studies in the region as evidenced by their application in Iraq, Qatar, and Saudi Arabia (25–27).

To bridge this gap in the literature, the current paper aims to describe the prevalence of mental health disorders, including probable depression, anxiety, and PTSD, in Lebanon and their associated correlates. Data was collected through telephone interviews using a nationally representative sample in the aftermath of the double assault of the Beirut Port Blast, the economic collapse during the prevailing COVID-19 pandemic. Although telephone interviewing as a research method is often met with skepticism, several studies have demonstrated its high reliability and effectiveness. When compared to traditional face-to-face interviews, telephone interviews have shown significant agreement in assessing various psychiatric disorders and mental health conditions (28–31). For instance, research has revealed excellent agreement for anxiety disorders, very good agreement for major depressive disorder, and alcohol/substance use disorders, and no significant differences in outcomes when using identical questionnaires for both telephone and face-to-face interviews (28, 32, 33).

## 2 Materials and methods

### 2.1 Sample

This study is a collaboration between IDRAAC, the research section of Saint George University Medical Center (SGUMC) and the global market research and consulting firm, Ipsos SAL. It aims to estimate the prevalence of mental health disorders and their associated factors within a nationally representative sample of Lebanese adults (18+) in the aftermath of the multiple co-occurring stressors in Lebanon including the Beirut Blast and Lebanese financial meltdown during the global pandemic. To achieve a nationally representative sample of the Lebanese population, we used the most recent Central Administration of Statistics report published by the Ministry of Public Health of Lebanon in 2019 which was completed in 1932 (34), to reach proportional representation of the gender and governorate within our sample. We utilized Ipsos’ proprietary database and employed the Computer Assisted Telephone Interview (CATI) system for the random selection of our representative sample. Accordingly, a total of 1,000 final participants were included in our study. Informed verbal consent was obtained before conducting interviews. These procedures were approved by the Institutional Review Board (IRB) committee of the Saint George Hospital University Medical Center, Faculty of Medicine, Lebanon. Grammar correction and language refinement for this manuscript were conducted using OpenAI's GPT-3.5, a generative AI technology accessed in January 2024 via the ChatGPT platform. The tool was employed solely to enhance the manuscript's clarity and readability.

### 2.2 Interviews

Interviews were held over the phone between July and September of 2022 by Ipsos’ team which come from a reputable outfit with a very long and well-established history of conducting phone interviews. To maintain meticulous record-keeping, all calls were systematically recorded, documenting interview outcomes, whether completed or not, responses or lack thereof, postponements, and other relevant metrics. Our study successfully interviewed a final sample of 1,000 participants, allowing for a margin of error of 5%. The average interview duration was approximately 15 minutes. Of the attempts made, 2,257 individuals did not respond to the call, 291 requested a callback, 611 encountered busy phone lines, 24 had their phones switched off, 168 declined to participate, and 6 exceeded the quota in case of no answer, three attempts were made before refraining from calling the number again. Additionally, 38 individuals did not complete the interviews, and 95 expressed a preference for a later appointment. Notably, 495 phone numbers were disconnected.

### 2.3 Enumerator training & quality control

Prior to data collection, enumerators and supervisors underwent rigorous training by Ipsos with regular feedback from IDRAAC. The program covered key aspects such as understanding study objectives, proficiency in administering components like the screener and questionnaire, familiarity with data collection protocols, adherence to ethical guidelines, strategies for respondent engagement, and effective handling of survey materials and equipment. To ensure data integrity, an internal quality control system which involved a systematic review of interview recordings to evaluate the quality and adherence to established protocols. Furthermore, to achieve external quality control, two researchers from IDRAAC reviewed a random subset of recordings using metrics that evaluated consistency (i.e., whether the interviewer read each question as it is), clarity (i.e., whether the interviewer repeated the question upon demand), and completeness (i.e., whether the interviewer read the introduction fully). The comprehensive revision confirmed the accuracy of the recordings and upheld the data integrity standards of our study.

### 2.4 Measurement

Our study builds upon the Ipsos international study that delved into public health concerns in 34 countries (35). While their investigation primarily addressed attitudes towards various government services, our team introduced additional questions encompassing demographic variables (age, gender, marital status, place of residence, and employment status) alongside an exploration of the impact of Lebanon’s economic situation on respondents. Moreover, we also included scales to evaluate the prevalence of probable PTSD secondary to the Beirut Port Blast, anxiety, and depression. The selection of questions underwent a meticulous process led by our research team. Below is a breakdown of the scales and scores added to the survey.

#### 2.4.1 PCL-5 (PTSD checklist for DSM-5)

To determine probable PTSD prevalence following the Beirut port explosion, we used the PTSD Checklist for DSM-5 (PCL-5), 20-item self-report questionnaire rating symptoms from 0 (not at all) to 4 (extremely) as per DSM-5 criteria (36). Symptoms rated moderately (2) or higher were endorsed. A DSM-5 proxy diagnosis requires meeting specific criteria: 1 item from Intrusion (B), 1 from Persistent Avoidance (C), 2 from Negative Alterations in Cognitions and Mood (D), and 2 from Arousal and Reactivity (E).

#### 2.4.2 GAD-7 (generalized anxiety disorder questionnaire)

To determine probable anxiety prevalence, we used the General Anxiety Disorder (GAD) questionnaire consisting of 7 items to evaluate recent symptoms. Each question received a rating from 0 (not at all) to 3 (nearly every day). Participants’ scores on the 7 questions were summed, resulting in a total score ranging from 0 to 21. Based on this continuous score, participants were categorized into two groups: those without anxiety (total score < 8) and those screening positive for anxiety (total score ≥ 8). This cutoff, as determined by Plummer et al. (37), has an 84% specificity and 83% sensitivity.

#### 2.4.3 PHQ-9 (patient health questionnaire for depression)

To determine the prevalence of probable depression, we used the PHQ-9, a 9-item tool that explores respondents’ experiences over the preceding two weeks. Each question is assigned a score from 0 to 3, where 0 signifies “not at all” and 3 denotes “nearly every day.” After summing the 9 items of PHQ-9, a total score ranging from 0 to 27 is obtained for each participant. A cut-off score of 10 was used for PHQ-9, which yields a specificity of 0.89 and a sensitivity of 0.85 (38).

#### 2.4.4 Composite financial score

To assess economic conditions, a composite score was derived from six variables, including income rank, monthly surplus, financial security, financial strain during gift-giving occasions, basic lifestyle changes, and leisure lifestyle changes. Variable codes were recoded to generate a composite score, with values ranging from 0 (favorable economic situation) to 17 (unfavorable economic situation). The variable codes were changed (recoded values are present in the **Supplementary Materials S1**).

### 2.5 Statistical analysis

The analyses conducted for this study were gender-specific, ensuring a nuanced exploration of outcomes. Frequencies and percentages were calculated for categorical variables, while means and standard deviations were computed for continuous variables. The bivariate level analysis involved Chi-squared tests for categorical variables and t-tests for continuous variables.

Following the initial analyses, multivariate analyses were conducted for each outcome variable, namely probable PTSD, depression, and anxiety, within the context of gender specificity. Variables showing significance at the bivariate level were included in the respective models. Additionally, 95% confidence intervals were calculated for these multivariate analyses, providing a range of plausible values for the observed effects. The analyses were performed using STATA version 13.

## 3 Results

### 3.1 Sample characteristics

**Table 1** provides a comprehensive overview of the socio-demographic characteristics of our study sample. Females constituted approximately half of the participants (51.3%), with a predominant presence in the 18 to 34-year age range (48.7%). The majority of individuals were married (Overall: 60.9%, Males: 70.3%, Females: 51.85%). Employment patterns varied between genders, with a higher percentage of employed men (78.19%) compared to employed women (36.06%). Geographically, Mount Lebanon had the highest representation at 44.4%, followed by the North (14.2%), the South (9.5%), Beirut (6.3%), and other regions. The total sample exhibited a mean composite financial score of 13.09 ± 3.53, with females scoring slightly higher (13.36 ± 3.11). For a detailed breakdown of the gender distribution of the sociodemographic features in our sample, please refer to **Table 1**.

**Table 1:** Descriptive statistics stratified by gender.

Measurements	Male (487, 48.7%)	Female (513, 51.3%)	Total (1000, 100%)	p-value
	N (%)	N (%)	N (%)	
Age				<0.001*
18-34	130 (26.69%)	250 (48.73%)	380 (38%)	
35-49	153 (31.42%)	114 (22.22%)	267 (26.7%)	
50-64	150 (30.8%)	99 (19.3%)	249 (24.9%)	
>65	54 (11.09%)	50 (9.75%)	104 (10.4%)	
Governorate				<0.001*
Beirut	27 (5.54%)	36 (7.02%)	63 (6.3%)	
Mount Lebanon	229 (47.02%)	215 (41.91%)	444 (44.4%)	
North	59 (12.11%)	83 (16.18%)	142 (14.2%)	
Akkar	19 (3.90%)	33 (6.43%)	52 (5.2%)	
Bekaa	45 (9.24%)	27 (5.26%)	72 (7.2%)	
Baalbek-Hermel	14 (2.87%)	41 (7.99%)	55 (5.5%)	
South	53 (10.88%)	42 (8.19%)	95 (9.5%)	
Nabatiyeh	41 (8.42%)	36 (7.02%)	77 (7.7%)	
Marital Status				<0.001*
Married	343 (70.43%)	266 (51.85%)	609 (60.9%)	
Not Married^t^	144 (29.57%)	247 (48.15%)	391 (39.1%)	
Employment Status				<0.001*
Employed^ŧ^	380 (78.19%)	185 (36.06%)	565 (56.56%)	
Unemployed^ŧŧ^	106 (21.81%)	328 (63.94%)	434 (43.44%)	
Composite Financial Score^ŧŧŧ^				0.014*
	12.81 ± 3.91	13.36 ± 3.11	13.09 ± 3.53	

t includes separated/widowed/divorced/never married.

^ŧ^ includes self-employed.

^ŧŧ^ includes retired/student/long-term sick or disabled/household duties.

^ŧŧŧ^ Mean ± standard deviation. (*) indicates significant p-value <0.05

It is important to point out that our study’s data closely mirrors the latest Lebanese population census published by the Central Administration of Statistics (34), which assures a representative snapshot of the country’s demographic at the level of governorate and gender separately. This alignment validates the broad applicability of our findings to the general Lebanese population.

### 3.2 Prevalence and comorbidities

Mental health disorders were prevalent among the study participants, as detailed in **Table 2**. Notably, 45.3% exceeded the threshold on the GAD-7 probable anxiety scale, showing gender disparities (49.12% of females and 41.27% of males; p-value=0.013). Similarly, 47.8% scored above 10 on the PHQ-9 depression scale, with variations between genders (51.46% of females and 43.94% of males; p-value=0.017). Furthermore, 43.5% met the probable diagnosis criteria for probable PTSD, with gender-specific prevalence (46.78% of females and 40.04% of males; p-value=0.032). These mental health disorders exhibited significant gender differences.

**Table 2:** Prevalence of mental health disorders by gender.

Measurements	Male (487, 48.7%)	Female (513, 51.3%)	Total (1000, 100%)	p-value
	N (%)	N (%)	N (%)	
Anxiety (GAD-7)				0.013*
No anxiety	286 (58.73%)	261 (50.88%)	547 (54.7%)	
GAD-7 anxiety	201 (41.27%)	252 (49.12%)	453 (45.3%)	
Depression (PHQ-9)				0.017*
No depression	273 (56.06%)	249 (48.54%)	522 (52.2%)	
PHQ-9 depression	214 (43.94%)	264 (51.46%)	478 (47.8%)	
PTSD (PCL-5)				0.032*
No PTSD	292 (59.96%)	273 (53.22%)	565 (56.5%)	
DSM-5 PTSD	195 (40.04%)	240 (46.78%)	435 (43.5%)	
Comorbidity				0.020*
No disorder	205 (42.09%)	167 (32.55%)	372 (37.2%)	
Any Disorder	282 (57.91%)	346 (67.45%)	628 (62.8%)	
One disorder	78 (16.02%)	93 (18.13%)	171 (17.1%)	
Two disorders	80 (16.43%)	96 (18.71%)	176 (17.6%)	
All disorders	124 (25.46%)	157 (30.6%)	281 (28.1%)	

(*) indicates significant p-value <0.05

**Table 2** also illustrates the intricate interplay of these disorders within the general sample. A substantial 62.8% of participants screened positive for at least one disorder. Notably, 28.10% screened positive for all three disorders, while 17.1% and 17.6% screened positive for one or two disorders alone, respectively.

For a detailed breakdown of comorbidities by gender, refer to **Table 2**.

### 3.3 Correlates at multivariate level

In the multivariate analysis (**Table 3**), we explored the factors associated with probable anxiety, depression, and PTSD across the genders, and our analysis yielded distinct patterns. Variables such as governorate, employment status, and marital status did not exhibit any significant associations with any of the mental health disorders.

**Table 3:** Multivariate analyses of anxiety, depression, and posttraumatic stress disorder within the sample population.

Measures	Anxiety^(a)^						Depression^(b)^						PTSD^(c)^					
	Males			Females			Males			Females			Males			Females		
	OR	95% CI	p-value	OR	95% CI	p-value	OR	95% CI	p-value	OR	95% CI	p-value	OR	95% CI	p-value	OR	95% CI	p-value
Age																		
18-34	1.00	…	…	1.00	…	…	1.00	…	…	1.00	…	…	1.00	…	…	1.00	…	…
35-49	0.84	(0.39, 1.81)	0.648	1.67	(0.82, 3.42)	0.160	1.03	(0.5, 2.14)	0.934	0.54	(0.27, 1.08)	0.082	1.29	(0.65, 2.55)	0.463	1.63	(0.91, 2.92)	0.100
50-64	0.50	(0.23, 1.11)	0.090	0.29	(0.15, 0.61)	0.001*	0.98	(0.46, 2.08)	0.956	1.41	(0.69, 2.87)	0.341	1.79	(0.9, 3.59)	0.099	1.94	(1.06, 3.53)	0.031*
>65	0.42	(0.15, 1.2)	0.104	0.45	(0.19, 1.09)	0.077	1.28	(0.48, 3.4)	0.618	1.26	(0.52, 3.04)	0.614	1.41	(0.57, 3.47)	0.457	1.10	(0.51, 2.38)	0.816
Marital Status																		
Married	1.00	…	…	1.00	…	…	1.00	…	…	1.00	…	…	1.00	…	…	1.00	…	…
Not Married^t^	0.89	(0.45, 1.77)	0.743	1.56	(0.91, 2.68)	0.107	0.96	(0.51, 1.8)	0.907	0.71	(0.42, 1.21)	0.211	1.25	(0.7, 2.23)	0.454	0.72	(0.46, 1.13)	0.156
Employment Status																		
Employed^ŧ^	1.00	…	…	1.00	…	…	1.00	…	…	1.00	…	…	1.00	…	…	1.00	…	…
Unemployed^ŧŧ^	1.29	(0.68, 2.47)	0.439	1.23	(0.72, 2.11)	0.457	1.87	(0.96, 3.61)	0.064	1.59	(0.94, 2.69)	0.085	1.09	(0.6, 1.97)	0.774	1.10	(0.69, 1.74)	0.700
Composite Financial Score																		
	1.14	(1.05, 1.24)	0.002*	1.12	(1.03, 1.22)	0.012*	1.14	(1.06, 1.23)	0.001*	1.12	(1.02, 1.22)	0.014*	1.07	(0.99, 1.15)	0.073	1.10	(1.02, 1.18)	0.015*
Governorate																		
Beirut	1.00	…	…	1.00	…	…	1.00	…	…	1.00	…	…	1.00	…	…	1.00	…	…
Mount Lebanon	2.16	(0.73, 6.37)	0.164	0.95	(0.33, 2.72)	0.930	0.53	(0.18, 1.58)	0.256	0.79	(0.3, 2.12)	0.640	0.47	(0.18, 1.24)	0.127	0.86	(0.36, 2.03)	0.727
North	1.23	(0.36, 4.18)	0.738	0.71	(0.23, 2.25)	0.566	0.99	(0.29, 3.33)	0.985	1.22	(0.4, 3.7)	0.723	0.86	(0.29, 2.61)	0.797	1.89	(0.72, 4.93)	0.195
Akkar	3.09	(0.6, 15.9)	0.178	0.78	(0.19, 3.15)	0.722	0.21	(0.04, 1.04)	0.056	1.28	(0.34, 4.84)	0.716	1.24	(0.3, 5.15)	0.763	0.92	(0.3, 2.83)	0.890
Bekaa	2.59	(0.72, 9.35)	0.147	0.59	(0.15, 2.31)	0.449	0.56	(0.15, 2.08)	0.388	1.19	(0.29, 4.87)	0.811	0.55	(0.17, 1.75)	0.309	2.5	(0.72, 8.63)	0.148
Baalbek-Hermel	2.67	(0.45, 15.86)	0.280	1.68	(0.46, 6.15)	0.433	0.24	(0.04, 1.57)	0.136	0.44	(0.12, 1.55)	0.201	0.88	(0.18, 4.18)	0.868	1.08	(0.36, 3.29)	0.891
South	2.95	(0.81, 10.69)	0.100	0.81	(0.23, 2.83)	0.742	0.29	(0.08, 1.07)	0.064	2.94	(0.84, 10.3)	0.092	0.46	(0.15, 1.47)	0.190	0.59	(0.21, 1.7)	0.328
Nabatieh	1.33	(0.36, 4.94)	0.670	0.76	(0.21, 2.8)	0.685	0.95	(0.25, 3.65)	0.942	1.36	(0.38, 4.91)	0.635	1.01	(0.31, 3.27)	0.991	1.38	(0.45, 4.21)	0.577

(a) controlling for depression and PTSD.

(b) controlling for anxiety and PTSD.

(c) controlling for depression and anxiety.

t includes separated/widowed/divorced/never married.

^ŧ^ includes self-employed.

^ŧŧ^ includes retired/student/long-term sick or disabled/household duties.

Age, on the other hand, showed a distinct association with probable anxiety and PTSD in females aged 50-64 compared to ages 18-34 (OR: 0.29, p-value=0.001; OR= 1.94, p-value=0.031 respectively) while remaining non-significant with other outcomes.

The financial composite score emerged as a consistent factor significantly associated with all three mental health disorders across genders, except for males with probable PTSD: probable anxiety (Males: OR= 1.14, p-value=0.002, Females: 1.12, p-value=0.012), probable depression (Males: OR= 1.14, p-value=0.001, Females: OR= 1.12, p-value=0.014), and probable PTSD (Males: OR= 1.08, p-value=0.073, Females: 1.10, p-value=0.015).

These nuanced findings suggest that, within the specific context of our study, geographic location, employment, marital status, and age may not hold substantial effect over the likelihood of experiencing mental health disorders.

### 3.4 Perceptions and outcomes

In the supplementary material, we present the results of the associations between participants’ perceptions and probable anxiety, depression, and PTSD while controlling for various relevant variables as shown in **Supplementary Table S2**. Nearly half the participants in the sample rate the quality of healthcare in the country as poor and very poor, and 54.8% believe that the quality of healthcare will get worse in the upcoming years. Most participants (65.1%) think about their mental wellbeing fairly and very often, and 28.5% think that mental health is more important than physical health.

For probable anxiety, perceiving physical health as more important than mental health at the level of the country was associated with a heightened risk (OR=1.59, p-value=0.032) when compared to perceiving the two as equally important.

For probable PTSD, none of the examined perceptions exhibited a significant association. Probable depression, on the other hand, demonstrated robust associations with various perceptions. Participants anticipating that the quality of healthcare will stay the same (OR=2.15, p-value=0.01) or get worse (OR=1.64, p-value=0.03) in the upcoming years, as opposed to improving, exhibited significant associations with probable depression. Similarly, those who reported thinking about mental well-being fairly often (OR=1.98, p-value=0.04) or very often (OR=1.98, p-value=0.03), in comparison to never thinking about it, were at an increased risk of probable depression. Conversely, believing that physical health is treated as more important than mental health at the level of the country was a protective factor for probable depression (OR=0.61, p-value=0.02) when compared to perceiving mental and physical health as equally important.

## 4 Discussion

This nationwide telephone interview study aimed to investigate the prevalence, comorbidity, and associated factors of mental health disorders particularly of probable anxiety, depression, and PTSD within a nationally representative sample from Lebanon. Conducted in the aftermath of cataclysmic events, most importantly, the Beirut Port Blast of 2020, a severe financial meltdown in Lebanon, and the COVID-19 pandemic, this study uncovers substantial rates of mental health disorders, indicating a considerable burden on the country’s mental health.

Our study revealed heightened rates of probable depression (47%), anxiety (45%), and PTSD (43%) within the general population. These figures are not surprising given the shocks experienced since 2020. Population shocks, including economic recessions, can increase the rates of mental health disorder (4). Our study’s results align with this evidence, revealing a significant association between deteriorating financial standing and depressive and anxiety disorders. This pattern is observable globally, including in regions such as Greece, Hong Kong, and others, where economic recessions correlated with elevated rates of anxiety and depressive disorders (10, 11, 39).

Moreover, recent reports from the Global Burden of Disease revealed that the estimates of depressive and anxiety disorders have increased in their global incidence (by an average of 27.6% and 25.6% respectively) as a result of COVID-19 pandemic and its ensuing social and economic repercussions (40). Peru serves as a good example, experiencing a fivefold increase in depression rates by 2020 compared to those reported in 2018 (17). This rise in global rates provides further context for the heightened prevalence rates of probable depression and anxiety observed in our sample.

However, when it comes to the high rates of probable PTSD in our sample, the story shifts. The rates observed in our sample do not compare to global estimates of PTSD—which often range between 0.5% and 14.5% across the general population who have suffered from trauma (41). Moreso, our rates do not compare to the rates of probable PTSD in exposed populations of China (17%) following similar Ammonium Nitrate explosions (7).

Our rates do compare to the reported rates across displaced populations of Ethiopians (40.8%), Sudanese (46%), Syrians (32%), and Iraqis (64%) — nations that have weathered multiple episodes of turmoil and unrest (42–44). This suggests a unique impact, potentially stemming from cumulative adversities experienced by the Lebanese population.

Historically, Lebanon’s lifetime prevalence of any mental disorder, as indicated by the LEBANON study (45), stood at 25.8%, falling within the World Mental Health (WMH) surveys’ normal range (12.0% to 47.4%, IQR: 18.1-36.1%) involving 85,052 respondents (46). Today, after the multiple cataclysmic events that have hit Lebanon, we observe an elevated rate of 62.8% for any disorder, surpassing our previous rates, further showcasing the shift in Lebanon’s mental health landscape.

Conventional determinants such as marital status, age, or governorate do not provide a clear explanation for our outcomes. The far-reaching impact of multiple traumas in Lebanon appear to affect individuals universally, irrespective of these typical determinants. A parallel can be drawn with a recent study by Atwoli conducted in South Africa, where conventional sociodemographic factors failed to explain trauma-related rates (47). Similarly, a study on Hurricane Katrina survivors in the US demonstrated that typical socio-economic and demographic associations with PTSD were not observed, highlighting the complex and pervasive nature of trauma’s impact (48).

This study is subject to several limitations, the foremost being the use of telephone interviews instead of traditional in-person household surveys due to financial constraints. It is noteworthy that this methodological choice is not unique to our study but is a pragmatic approach seen in various national studies around the world, validating its reliability in the literature (11, 49, 50). Additionally due to logistical challenges, we were unable to include detailed exposure questions and comprehensive inquiries into participants’ previous mental health history or treatment-seeking patterns. Particularly, we did not ask participants about their substance use which often increases during time of crises.

In conclusion, this study represents Lebanon’s first national study to be conducted on mental health disorders since the WMH in 2006 (24). Our findings revealed elevated rates of mental health disorders in Lebanon, namely probable depression, anxiety, and PTSD till present. There has been a shift since 2006, where low treatment-seeking rates were observed (45). Today, our Lebanese sample shows a substantial 65% recognizing mental health disorders as primary concerns – in contrast to the American Psychiatric Association’s report of only 25% prioritizing mental health in the U.S. by 2024 (51). This growing openness to seeking help for mental health issues underscores the importance of government action to provide comprehensive support at a national level. Furthermore, this study contributes valuable insights into mental health dynamics in Low- and Middle-Income Countries, filling a crucial gap in the existing literature.

## Data Availability

The datasets presented in this study can be found in online repositories. The names of the repository/repositories and accession number(s) can be found below: https://doi.org/10.6084/m9.figshare.25974934.v1.
