# Supplementary material for: The aftermath of multiple trauma on a nation: unraveling Lebanon’s unique mental health struggle
Source: Front Psychiatry. 2025 Jan 14;15:1444245. doi: 10.3389/fpsyt.2024.1444245 (PMC11773410; doi:10.3389/fpsyt.2024.1444245)
Supplement: Supplementary file 1 [file Table1.docx]

Supplementary Material

# Steps to generate the financial composite score

Six variables were considered **(D3-Income rank, D5-financial security for the next 2 years, D7-Strain on finance if buying a gift for occasions, D4-Money left over at the end of the month, Q8-basic lifestyle changes and Q9-Leisure lifestyle changes)** and each one was recoded. The recode process is described in detail in the table below, with values of 0 indicating a good outcome (as per Dr. Karam’s choice) and values of 3-4 indicating an unfavorable outcome.

Table S1 Scores of variable items that make up the financial composite score

| **Variables** | **Options** | **Score** |
| --- | --- | --- |
| Income Rank | Lower Class | 4 |
|  | Between Lower and Middle Class | 3 |
|  | Middle Class | 2 |
|  | between Middle and Higher class | 1 |
|  | Upper class | 0 |
| Financial security for the next 2 years | Completely | 0 |
|  | Somewhat | 1 |
|  | Not at all | 2 |
| Strain on finances if buying for gifts for occasions | Completely | 2 |
|  | Somewhat | 1 |
|  | Not at all | 0 |
| Money left over at the end of the month | I always have money left over | 0 |
|  | I always have enough money with little extra | 1 |
|  | I have just enough, no more | 2 |
|  | I cannot make ends meet | 3 |
| Basic lifestyle changes | Not at all | 0 |
|  | A little | 1 |
|  | Moderately | 2 |
|  | A lot | 3 |
| Leisure lifestyle changes | Not at all | 0 |
|  | A little | 1 |
|  | Moderately | 2 |
|  | A lot | 3 |

Then, these 6 recoded variables were added up together to generate the composite score ranging from 0 to 17

| Table S2. Perceptions association with anxiety, depression, and PTSD | | | | | | | | | | | |
| --- | --- | --- | --- | --- | --- | --- | --- | --- | --- | --- | --- |
|  | |  | **Anxiety^(a)^** | | | **Depression^(b)^** | | | **PTSD^(c)^** | | |
| **Measures** | |  | **OR** | **p-value** | **(95% CI)** | **OR** | **p-value** | **(95% CI)** | **OR** | **p-value** | **(95% CI)** |
| **Rate the quality of healthcare you and your family have access to in your country** | | | | | |  |  |  |  |  |  |
| Very Good | | 67 (6.7%) | 1.00 | … | … | 1.00 | … | … | 1.00 | … | … |
| Good | | 223 (22.3%) | 0.74 | 0.481 | (0.33, 1.7) | 1.09 | 0.85 | (0.47, 2.53) | 0.99 | 0.97 | (0.48, 2.03) |
| Neither Good nor Poor | | 199 (19.9%) | 1.12 | 0.784 | (0.49, 2.59) | 0.60 | 0.25 | (0.25, 1.43) | 1.29 | 0.50 | (0.62, 2.68) |
| Poor | | 182 (18.2%) | 1.11 | 0.806 | (0.48, 2.6) | 0.82 | 0.66 | (0.34, 1.99) | 1.26 | 0.55 | (0.6, 2.65) |
| Very Poor | | 329 (32.9%) | 1.29 | 0.543 | (0.57, 2.88) | 0.71 | 0.44 | (0.31, 1.67) | 1.35 | 0.40 | (0.67, 2.73) |
| **Will the quality of healthcare in your country change in the upcoming years?** | | | | | |  |  |  |  |  |  |
| It will improve | | 289 (28.9%) |  |  |  | 1.00 | … | … |  |  |  |
| It will stay the same | | 163 (16.3%) |  |  |  | 2.15 | 0.01* | (1.2, 3.85) |  |  |  |
| It will get worse | | 548 (54.80%) |  |  |  | 1.64 | 0.03* | (1.05, 2.54) |  |  |  |
| **How often do you think about mental wellbeing?** | | | |  |  |  |  |  |  |  |  |
| Never | | 176 (17.65%) | 1.00 | … | … | 1.00 | … | … | 1.00 | … | … |
| Not very often | | 172 (17.25%) | 0.86 | 0.662 | (0.44, 1.68) | 1.29 | 0.45 | (0.67, 2.49) | 1.02 | 0.94 | (0.58, 1.8) |
| Fairly Often | | 197 (19.76%) | 0.79 | 0.487 | (0.4, 1.54) | 1.98 | 0.04* | (1.02, 3.84) | 0.83 | 0.54 | (0.47, 1.49) |
| Very Often | | 452 (45.34%) | 1.27 | 0.444 | (0.69, 2.32) | 1.98 | 0.03* | (1.09, 3.62) | 1.15 | 0.60 | (0.68, 1.92) |
| **How often do you think about your physical wellbeing?** | | | | |  |  |  |  |  |  |  |
| Never | | 131 (13.11%) | 1.00 | … | … | 1.00 | … | … | 1.00 | … | … |
| Not very often | | 147 (14.71%) | 0.94 | 0.862 | (0.45, 1.95) | 0.86 | 0.70 | (0.41, 1.82) | 0.91 | 0.77 | (0.48, 1.71) |
| Fairly Often | | 259 (25.93%) | 1.11 | 0.774 | (0.55, 2.23) | 0.43 | 0.02* | (0.21, 0.86) | 0.77 | 0.39 | (0.43, 1.39) |
| Very Often | | 462 (46.25%) | 0.92 | 0.797 | (0.48, 1.76) | 0.63 | 0.17 | (0.32, 1.22) | 1.00 | 0.99 | (0.58, 1.74) |
|  | | | | | |  |  |  |  |  |  |
| **What do you personally think is more important, mental health or physical health?** | | | | | |  |  |  |  |  |  |
| Equally Important | | 593 (59.30%) | 1.00 | … | … |  |  |  |  |  |  |
| Mental health is more important than physical health | | 285 (28.5%) | 1.29 | 0.252 | (0.83, 1.99) |  |  |  |  |  |  |
| Physical health is more important than mental health | | 122 (12.2%) | 0.98 | 0.940 | (0.54, 1.78) |  |  |  |  |  |  |
| **At the level of your country, what is more important mental health or physical health?** | | | | | | |  |  |  |  |  |
| Equally Important | | 334 (35.53%) | 1.00 | … | … | 1.00 | … | … | 1.00 | … | … |
| Mental health is treated as more important than physical health | | 137 (14.57%) | 1.77 | 0.057 | (0.98, 3.19) | 0.97 | 0.93 | (0.54, 1.75) | 1.10 | 0.70 | (0.67, 1.81) |
| Physical health is treated as more important than mental health | | 469 (49.89%) | 1.59 | 0.032* | (1.04, 2.42) | 0.61 | 0.02* | (0.41, 0.92) | 0.78 | 0.18 | (0.55, 1.12) |
|  | (a) controlling for sex, age, marital status, employment status, financial composite score, PTSD, and depression | | | | | | | | |  |  |
|  | (b) controlling for sex, governorate, marital status, employment status, financial composite score, PTSD, and anxiety | | | | | | | | | |  |
|  | (c) controlling for sex, age, governorate, marital status, employment status, financial composite score, depression, and anxiety | | | | | | | | | | |
